# Differences in the Rates of Diagnoses of Mental and Behavioral Disorders Due to Psychoactive Substance Use by Sex and Age during Pre-Pandemic and COVID-19 Pandemic Periods in Kazakhstan

**DOI:** 10.3390/healthcare12202012

**Published:** 2024-10-10

**Authors:** Kamila Akkuzinova, Ken Inoue, Elaman Toleuov, Timur Moldagaliyev, Nursultan Seksenbayev, Ulzhan Jamedinova, Nargul Ospanova, Altay Dyussupov

**Affiliations:** 1 Board, Semey Medical University, Semey 071400, Kazakhstan; akkuzinova@gmail.com (K.A.); elamantol96@gmail.com (E.T.); timur_party@inbox.ru (T.M.); nurs_7sk@inbox.ru (N.S.); nargul_ospanova@mail.ru (N.O.); 2 Research and Education Faculty, Medical Sciences Cluster, Health Service Center, Kochi University, Kochi 780-8520, Japan; 3 Department of Biostatistics and Epidemiology, Semey Medical University, Semey 071400, Kazakhstan; ulzhan.jamedinova@nao-mus.kz; 4 Board, Semey Medical University, Semey 071400, Kazakhstan; altay.dyusupov@nao-mus.kz

**Keywords:** mental and behavioral disorders due to psychoactive substance use, COVID-19 pandemic, age, male, female, difference, Kazakhstan

## Abstract

Background: The COVID-19 pandemic had profound impacts worldwide on individuals with mental and behavioral disorders, including disorders due to psychoactive substance use. We investigated how the COVID-19 pandemic affected the trends in these disorders in the Republic of Kazakhstan. Methods: We researched and compared ICD-10 data on mental and behavioral disorders due to substance use in Kazakhstan that were diagnosed in 2018–2019 (pre-pandemic) versus 2020–2021 (the pandemic period). Results: The data for the pandemic period were significantly different from those of the pre-pandemic in that (*i*) ‘other stimulant-related disorders (F15)’ and ‘other psychoactive substance-related disorders (F19)’ were increased in the younger age groups, (*ii*) the risk of ‘opioid-related disorders (F11)’ was decreased in the 30-year-old group in both males and females, and (*iii*) the risk of ‘alcohol-related disorders (F10)’ was increased in the 30-year-old group and decreased in the 20- and 50-year-old groups. In only the males, (*iv*) the risk of ‘other psychoactive substance-related disorders (F19)’ was increased in almost all of the age groups, and (*v*) the risk of ‘cannabis-related disorders (F12)’ was increased in the ≥50-year-olds. The pre-pandemic and pandemic periods thus involved changes due to COVID-19 in both males and females that were especially notable in males. Conclusions: These results indicate that further measures designed to prevent mental and behavioral disorders due to psychoactive substances are necessary at the primary, secondary, and tertiary levels, and personnel in medicine/nursing, the government, private organizations, and the public need to collaborate to devise such measures.

## 1. Introduction

The International Classification of Diseases, 10th revision (ICD-10), issued by the World Health Organization (WHO) refers to a variety of conditions associated with the excessive use of psychoactive substances, including alcohol, narcotics, and prescription medications, as “mental and behavioral disorders due to psychoactive substance use”; the 11th revision (ICD-11) refers to them as “disorders due to substance use”. One of the features of these conditions is the inability to quit using psychoactive substances despite their negative effects, which significantly hinders daily functioning and health [1,2]. These conditions are referred to as “substance use disorders” in the Diagnostic and Statistical Manual of Mental Disorders, Fifth Edition (DSM-5), issued by the American Psychiatric Association (APA) [3].

The ICD-10 classifies mental and behavioral disorders due to psychoactive substance use based on the psychoactive substance taken; that is, they are categorized as those due to use of alcohol (F10: alcohol-related disorders), opioids (F11: opioid-related disorders), cannabinoids (F12: cannabis-related disorders), sedatives or hypnotics (F13: sedative, hypnotic, or anxiolytic-related disorders), cocaine (F14: cocaine-related disorders), other stimulants including caffeine (F15: other stimulant-related disorders), hallucinogens (F16: hallucinogen-related disorders), tobacco (F17: nicotine dependence), volatile solvents (F18: inhalant-related disorders), and multiple drug use and the use of other psychoactive substances (F19: other psychoactive substance-related disorders) [1].

In 2019, the worldwide prevalence of disorders due to substance use was 2.2% of the global population, with alcohol use disorders accounting for 1.5% at that time [4]. In 2016, approx. 2.3 billion people were consumers of alcohol globally, and although the overall population’s prevalence of heavy episodic drinking declined from 22.6% in 2000 to 18.2% in 2016, this prevalence remains high among drinkers, particularly in some Eastern European and sub-Saharan African countries [5].

According to the World Drug Report 2020, 4.8% of the global general population reported having used illegal substances at some point in their lives in 2009 [6], and by 2018, the percentage of persons using narcotics had climbed by 0.5%, with the growth being greater in developing countries [6]. Among individuals between the ages of 15 and 65, the prevalence of disorders related to the use of illicit drugs was 0.6% in 2016 and slightly higher, at 0.7%, in 2019 [7]. According to a study that analyzed data from the Institute of Health Metrics and Evaluation database for 2019, the global prevalence of disorders due to substance use was 2.2% [4]. The World Drug Report 2017 demonstrated that among illegal drugs, cannabinoids (3.8%), amphetamines (0.77%), opioids (0.37%), and cocaine (0.35%) were the most commonly used drugs by individuals aged 15–64 years; cannabis dependence in 2015 was identified in 0.26% of the general population, opioid dependence in 0.22%, and amphetamine and cocaine dependence in 0.086% and 0.052% [8].

The countries of the former Soviet Union with the highest tobacco smoking rates among males were Armenia and Georgia [9]. The Republic of Kazakhstan (hereafter, Kazakhstan) and Japan showed similar trends between the sexes [9]. Despite the global decline in the incidence of tobacco use during the 30 years from 1990 to 2019, men in countries such as Afghanistan, Uzbekistan, Mongolia, and Indonesia became more likely to smoke tobacco, whereas women in Kyrgyzstan, Afghanistan, Mongolia, Belarus, and Russia were also more likely to smoke tobacco [9].

In December 2019, an outbreak of pneumonia of unknown etiology was reported in the city of Wuhan in China and was later confirmed to be caused by a novel coronavirus. The rapid spread of the infection worldwide led the WHO to declare the SARS-CoV-2 infection outbreak as a pandemic on 11 March 2020 [10]. As with many global crises facing humanity, the COVID-19 pandemic had an undeniable impact on individuals with mental and behavioral disorders, including disorders due to psychoactive substance use. The pandemic put additional pressure on healthcare systems and led to economic downturns and social challenges worldwide. Public health was greatly influenced by the COVID-19 pandemic, which increased existing health inequities and brought new difficulties [11,12]. The pandemic led to an increase in global mortality that is not only due to the direct impacts of the virus on people’s health. By the end of 2021, COVID-19 deaths had reached 5.94 million, with excess mortality during this period exceeding 18 million, which is more than three times the number of deaths directly caused by the SARS-CoV-2 virus [13].

The pandemic itself and the social distancing measures used to control the spread of infection have been traumatic for many people and were accompanied by an increase in the prevalence of mental disorders [13,14]. A study conducted in China revealed a high proportion of post-traumatic stress disorder among patients hospitalized for SARS-CoV-2 infection [15]. All populations have experienced markedly higher levels of stress and anxiety as the result of the pandemic [14,16], and a pandemic-related worsening of the conditions of patients with pre-existing mental disorders was reported [14,16,17].

Due to the frequent co-occurrence of mental and physical health conditions, individuals who have substance use disorders are especially susceptible to health emergencies such as the COVID-19 pandemic [14]. Several studies suggest positive correlations between substance use disorders and anxiety-, depressive-, and stress-related disorders [18,19,20]. Impacts of the pandemic on the prevalence of substance use disorders—particularly factors such as isolation and stress caused by quarantine measures—have also been described [21,22]. According to a study assessing the effects of social distancing during the initial months of COVID-19 in the United States, drug users with more severe drug habits reported feeling more socially isolated, and increased alcohol and tobacco intakes were linked to this isolation [22].

The pattern of change in the prevalence of substance consumption during the COVID-19 pandemic showed a highly variable and complex trend, reflecting diverse contextual influences and underlying conditions. This variability indicated that the impact of the pandemic on substance use behaviors cannot be easily generalized and was shaped by a range of unique circumstances, making it challenging to identify a consistent global trajectory [23]. A cross-sectional study in Germany discovered shifts in substance usage [24]. According to a study conducted in Japan, alcohol use increased during the pandemic compared to pre-pandemic among both males and females [25].

Due to the influence of drug use on the progression of SARS-CoV-2 infection, research on substance use disorders in relation to the COVID-19 pandemic is especially crucial. According to a U.S. study performed by Wang et al., individuals with a substance use disorder have a higher risk of COVID-19 infection than the general population. The most vulnerable were individuals addicted to opioids [26].

Due to quarantine/lockdown regulations, unstable economic conditions, and the need to relocate medical personnel in order to combat infection, the pandemic significantly impacted the availability of medical services for people with substance use disorders [27,28,29,30]. These changes have exacerbated pre-existing barriers, such as health disparities, lack of access to resources, and regulatory constraints, which have disproportionately impacted marginalized communities [31]. In many low- and middle-income countries, patients with mental problems or disorders due to substance use experienced difficulty receiving care during the pandemic due to problems such as the cancelation of in-person consultations, the transfer of mental health professionals to treat patients infected with SARS-CoV-2, and the public’s anxiety over being infected with SARS-CoV-2 during healthcare visits [32]. All of these factors increase the likelihoods of overdose and relapse in persons who use psychoactive substances. A surge in emergency department visits due to opioid overdoses during the pandemic, along with an increased count of opioid-associated fatalities compared to the pre-pandemic period, have been documented [33,34]. Opioid-related deaths have also increased, notably those involving synthetic opioids such as fentanyl [35]. Opioid-related overdose deaths were reported to be highest in individuals aged 30–40 years old [36]. One piece of research suggested that individuals with substance use disorders were particularly vulnerable due to co-occurring comorbidities and adverse social determinants of health, which necessitate adaptive healthcare strategies that can accommodate these complex needs [37].

Limited data on COVID-19 and substance use problems in low- and middle-income countries (which include, inter alia, the countries of Central Asia) have been reported, necessitating further research [28]. The scarcity of investigations focusing on the intersection of the COVID-19 pandemic and mental and behavioral disorders resulting from psychoactive substance use in these regions is notable. In Kazakhstan, there have been few investigations into how the pandemic has impacted these disorders. Major issues regarding mental and behavioral disorders due to psychoactive substance use and the influences of the pandemic arose in Kazakhstan as elsewhere [38,39,40], and we thus conducted the present study to address these issues and their resolution by performing analyses of data concerning individuals with mental and behavioral disorders due to psychoactive substance use that were collected before and during the COVID-19 pandemic.

By examining the changes in these disorders over the described period, we hope to shed light on the pandemic’s impact on substance use in Kazakhstan. Our findings contribute to the limited body of knowledge and underscore the importance of addressing substance use problems in the context of global health crises, particularly in low- and middle-income countries such as Kazakhstan. There have been few standardized-format studies of the relationships between substance use problems and the COVID-19 pandemic in Kazakhstan, and the present study of these relationships in Kazakhstan was based on formats used worldwide.

### The Importance of This Study

- Detailed effects of the COVID-19 pandemic on individuals with mental or behavioral disorders due to their use of psychoactive substances have been unclear, not only in Kazakhstan but also globally.
- Very few studies have examined the combination of these topics in detail, and the present investigation is novel in its use of conceptual and contextual viewpoints.
- Our comparison of the status of mental and behavioral disorders due to psychoactive substance use before and during the COVID-19 pandemic also uses a novel methodological viewpoint.

## 2. Materials and Methods

### 2.1. Study Design

The study’s design was retrospective. We analyzed the data of mental and behavioral disorders due to substance use that were diagnosed according to the ICD-10 criteria, including disorders due to use of ‘alcohol (F10)’, ‘opioids (F11)’, ‘cannabinoids (F12)’, ‘sedatives and hypnotics (F13)’, ‘cocaine (F14)’, ‘other stimulants including caffeine (F15)’, ‘hallucinogens (F16)’, ‘volatile solvents (F18)’, and ‘multiple drug use and the use of other psychoactive substances (F19)’. We excluded the data of disorders due to the use of ‘tobacco (F17)’ because of a lack of these data before the publication timepoint used herein. The data of the diagnoses in 2018–2019 (i.e., during the pre-pandemic period) and those in 2020–2021 (during the COVID-19 pandemic) were compared.

We divided the subjects into the following age groups: ≤19, 20–29, 30–39, 40–49, 50–59, and ≥60 years old. We analyzed the data of the total population, the six age groups, and males and females separately. We used only numerical data without the individual subjects’ information.

### 2.2. Data Collection

We obtained anonymized data on individuals in Kazakhstan diagnosed with mental and behavioral disorders due to substance use, classified according to the ICD-10 criteria (codes F10–F19) from the Republican Scientific and Practical Centre of Mental Health of the Republic of Kazakhstan. The data were collected for the period from 2018 to 2021, covering the entire country and ensuring national representativeness. These data are compiled annually from reports submitted by all organizations providing mental health care in the Republic of Kazakhstan.

The initial dataset contained information on all patients diagnosed with any mental or behavioral disorder due to substance use within the specified period. It included complete demographic details, including age and sex, for each patient. Consequently, no exclusions based on demographic characteristics were required at the pre-processing stage. Moreover, since all diagnoses in the registry were confirmed at the time of their documentation, there were no unverified or repeated entries, ensuring that the data were free of duplicates and incomplete records.

The total number of cases meeting the study’s inclusion criteria amounted to 471,807 individuals with substance use disorders. The final sample for analysis thus consisted of 471,807 confirmed and unique records accurately reflecting the target population within the defined period.

### 2.3. Statistical Analysis

We divided the data into two periods: the pre-pandemic period, i.e., the data from the years 2018–2019, and the pandemic period, i.e., the data from 2020 to 2021. We compared the number of new diagnoses of each mental and behavioral disorder due to substance use between these two periods using odds ratios (OR) and 95% confidence intervals (CIs). The sex and age-group data were also compared with ORs and 95%CIs. An OR > 1 indicated an increase in the number of new diagnoses of mental and behavioral disorders due to substance use during the pandemic years, and an OR < 1 indicated a decrease.

Although the proportion of diagnoses differed between the males and females (84.74% for males and 15.26% for females in the pre-pandemic period and 84.54% for males and 15.46% for females in the pandemic period), the absolute number in both groups was sufficiently large to allow for meaningful statistical analyses. Specifically, during the pre-pandemic period, there were 209,066 males and 37,656 females, and in the pandemic period, there were 190,293 males and 34,792 females. These sample sizes ensured adequate statistical power to draw reliable conclusions for both sexes.

However, it is important to note that although the dataset provided information on diagnoses, sex, and age, it lacked data on potential confounding factors such as socioeconomic status, access to healthcare, and regional differences. As a result, these variables could not be controlled for in the analyses, and this may have influenced the observed associations between the pandemic and the patterns in substance use disorders.

Probability (*p*)-values < 0.05 were accepted as significant. All statistical analyses were performed using R Statistical Software (ver. 4.4.1, R Core Team 2024, Vienna, Austria).

## 3. Results

There was a total of 471,807 diagnoses of mental and behavioral disorders due to substance use in Kazakhstan during the 4-year study period. Our analyses revealed an overall decrease in this number during the pandemic period compared to the pre-pandemic period (from 246,722 to 225,085 instances); similar decreases were observed for the males and the females. Males and females, respectively, accounted for 84.74% and 15.26% of the diagnoses during the pre-pandemic period and for 84.54% and 15.46% in the pandemic period.

**Figure 1:**
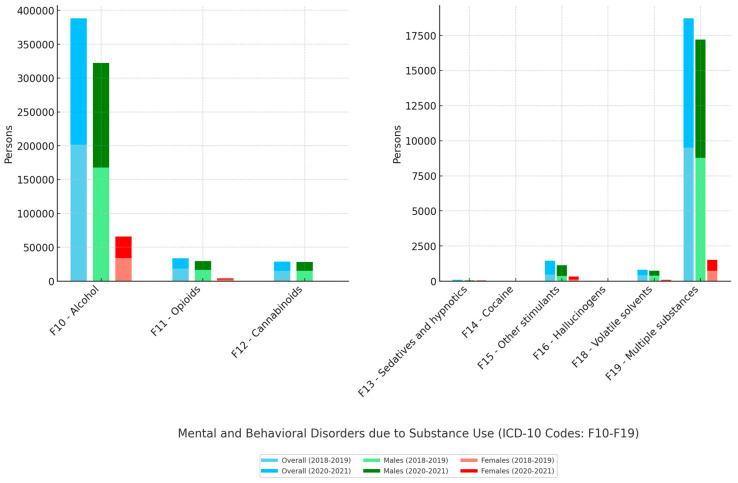
The number of new diagnoses of mental and behavioral disorders due to substance use in Kazakhstan during the pre-pandemic period (2018–2019) and the COVID-19 pandemic (2020–2021) in the overall population, males, and females.

The distributions of the diagnoses of mental and behavioral disorders due to substance use by age group were as follows: 0.60% pre-pandemic and 0.45% pandemic in the ≤19-year-old age group, 8.15% and 6.49% in the 20–29 group, 27.16% and 25.34% in the 30–39 group, 31.77% and 32.81% in the 40–49 group, 21.46% and 22.16% in the 50–59 group, and 10.87% and 13.16% in the ≥60 group, respectively. Most of the diagnoses were disorders due to the use of alcohol, with a total of 201,971 during the pre-pandemic period and 186,137 during the pandemic; among males, the corresponding numbers were 167,717 and 154,494, and the corresponding numbers among females were 34,254 and 31,643. The data of each mental and behavioral disorder due to substance use for the overall population, males, and females are depicted in Figure 1.

### 3.1. Trends for the Overall Group

Table 1 summarizes the results of our analyses of the diagnoses of mental and behavioral disorders due to substance use during the pre-pandemic and pandemic periods for the overall population. In only the ≥60-years age group, the number of these diagnoses increased from the pre-pandemic period to the pandemic period.

‘Alcohol’ was the most frequent diagnosis in all age groups (all ages, ≤19, 20–29, 30–39, 40–49, 50–59, and ≥60), as follows: pre-pandemic, ‘alcohol’ was the diagnosis for 81.86%, 60.39%, 59.02%, 74.90%, 81.66%, 92.38%, and 97.41% of the total, respectively; during the pandemic, ‘alcohol’ was the diagnosis for 82.70%, 58.79%, 57.12%, 75.92%, 81.74%, 91.55%, and 97.00%, respectively.

In our comparison of the pre-pandemic and pandemic periods in the overall population, including all age groups, the pandemic period data showed significantly higher odds compared to the pre-pandemic period for ‘alcohol’, ‘other stimulants including caffeine’, and ‘multiple drug use and the use of other psychoactive substances’. Significantly lower odds were observed in the pandemic period compared to the pre-pandemic period for ‘opioids’ and ‘cannabinoids’.

In the ≤19-years age group, significantly higher odds during the pandemic were revealed for ‘other stimulants including caffeine’ and ‘multiple drug use and the use of other psychoactive substances’ compared to the pre-pandemic results. In contrast, the pandemic period exhibited significantly lower odds in this age group for ‘cannabinoids’ versus the pre-pandemic data.

In the 20–29 age group, the pandemic showed significantly higher odds for ‘other stimulants including caffeine’ and ‘multiple drug use and the use of other psychoactive substances’ and significantly lower odds for ‘alcohol’ and ‘cannabinoids’ compared to the pre-pandemic period.

In the 30–39 group, significantly higher odds were shown for the pandemic compared to the pre-pandemic period regarding ‘alcohol’, ‘cannabinoids’, ‘other stimulants including caffeine’, and ‘volatile solvents’ and significantly lower odds regarding ‘opioids’.

In the 40–49 group, significantly higher odds for diagnoses of ‘other stimulants including caffeine’ and ‘multiple drug use and the use of other psychoactive substances’ were observed in the pandemic period versus the pre-pandemic years. The pandemic years showed significantly lower odds for ‘opioids’ in this group compared to before the pandemic.

In the subjects aged 50–59 years, the pandemic data showed significantly higher odds for ‘opioids’, ‘cannabinoids’, and ‘multiple drug use and the use of other psychoactive substances’ than the pre-pandemic data. An ‘alcohol’ diagnosis was associated with significantly lower odds during the pandemic compared to the pre-pandemic data.

In the group aged ≥60 years, the pandemic years showed significantly higher odds for ‘cannabinoids’ and ‘multiple drug use and the use of other psychoactive substances’ and significantly lower odds for ‘alcohol’ diagnoses versus the pre-pandemic years.

### 3.2. Trends for the Males

Table 2 presents the results of the analyses of mental and behavioral disorders due to substance use during the pre-pandemic and COVID-19 pandemic periods among only males. In only the group of males aged ≥60 years, the rate of diagnoses was greater during the pandemic compared to the pre-pandemic period.

‘Alcohol’ was the most frequent diagnosis in each age group of males (all ages, ≤19, 20–29, 30–39, 40–49, 50–59, and ≥60 years) as follows: pre-pandemic, ‘alcohol’ was the diagnosis for 80.22%, 56.14%, 56.49%, 73.04%, 80.06%, 91.55%, and 97.18% of the total; during the pandemic, it was the diagnosis for 81.19%, 53.54%, 55.17%, 74.02%, 80.21%, 90.71%, and 96.73%.

For the overall group of males (including all of the age groups), the pandemic years showed significantly higher odds for ‘alcohol’, ‘other stimulants including caffeine’, and ‘multiple drug use and the use of other psychoactive substances’ and significantly lower odds for ‘opioids’ and ‘cannabinoids’ compared to the pre-pandemic years.

In the ≤19-years group of males, the pandemic was associated with significantly higher odds for ‘multiple drug use and the use of other psychoactive substances’ versus the pre-pandemic period.

In the 20- to 29-year-old males, significantly higher odds were observed for ‘other stimulants including caffeine’ and ‘multiple drug use and the use of other psychoactive substances’ and significantly lower odds for ‘alcohol’ and ‘cannabinoids’ during the pandemic compared to the pre-pandemic period.

In the group of subjects aged 30–39 years, the pandemic period was linked to significantly higher odds for diagnoses of ‘alcohol’, ‘cannabinoids’, ‘other stimulants including caffeine’, and ‘volatile solvents’ disorders compared to the pre-pandemic data, with significantly lower odds for disorders associated with ‘opioids’.

In the 40–49 age group, the pandemic was associated with significantly higher odds for ‘other stimulants including caffeine’ and ‘multiple drug use and the use of other psychoactive substances’ and significantly lower odds for ‘opioids’ compared to the pre-pandemic period.

In the males aged 50–59, the COVID-19 pandemic data revealed significantly higher odds for ‘cannabinoids’, and ‘multiple drug use and the use of other psychoactive substances’, and significantly lower odds for ‘alcohol’ compared to the pre-pandemic data.

In the males ≥60 years, compared to the pre-pandemic period, the COVID-19 pandemic was associated with significantly higher odds for ‘cannabinoids’ and ‘multiple drug use and the use of other psychoactive substances’ and significantly lower odds for ‘alcohol’ than pre-pandemic.

### 3.3. Trends for the Females

The results of our analyses of the diagnoses of mental and behavioral disorders due to substance use during the pre-pandemic and COVID-19 pandemic periods among female participants are presented in Table 3. The number of these diagnoses increased from the pre-pandemic period to the pandemic period only in females aged ≥60 years.

‘Alcohol’ was also the most frequent in each age group of females (all ages, ≤19, 20–29, 30–39, 40–49, 50–59, and ≥60), as follows: pre-pandemic, ‘alcohol’ was the diagnosis for 90.97%, 75.54%, 79.75%, 86.09%, 90.29%, 96.67%, and 98,46% of the total; during the pandemic, it was the diagnosis for 90.95%, 74.03%, 71.38%, 87.53%, 89.97%, 95.89%, and 98.26%.

The comparison of the pre-pandemic and pandemic periods in the overall group of females (including all of the age groups) revealed that the pandemic was associated with significantly higher odds for ‘other stimulants including caffeine’ and ‘multiple drug use and the use of other psychoactive substances’ and significantly lower odds for ‘opioids’ versus the pre-pandemic period.

Among females aged ≤19 years, the COVID-19 pandemic data showed significantly lower odds for ‘cannabinoids’.

In the 20–29 age group, the pandemic was associated with significantly higher odds for ‘other stimulants including caffeine’ and ‘multiple drug use and the use of other psychoactive substances’ and significantly lower odds for ‘alcohol’ compared to the pre-pandemic years.

In females at 30–39 years old, significantly higher odds were observed for ‘alcohol’, ‘cannabinoids’, and ‘other stimulants including caffeine’ and significantly lower odds for ‘opioids’ during the pandemic versus the pre-pandemic.

Females aged 50–59 years exhibited significantly higher odds for ‘opioids’ and significantly lower odds for ‘alcohol’ during the pandemic versus the pre-pandemic years.

In the two groups of females aged 40–49 and ≥60, we observed no significant differences in the numbers of diagnoses made during the pre-pandemic and pandemic periods.

## 4. Discussion

Our comparisons of the mental and behavioral disorders due to substance use diagnosed in Kazakhstan during a two-year pre-pandemic period and the two-year COVID-19 pandemic obtained the following main findings. Among the total of 471,807 diagnoses of mental and behavioral disorders due to psychoactive substance use across all of the age groups combined, the pandemic was marked by increases in disorders related to alcohol, other stimulants including caffeine, and multiple drug use and the use of other psychoactive substances in particular. In contrast, the results for opioid and cannabinoid-related disorders indicated that more attention should be paid to these ICD-10 codes during the pre-pandemic years compared to the pandemic. These trends both differed between males and females and showed common aspects between the sexes.

The commonalities in the males/females were that the pandemic was significantly associated with increased risks of disorders linked to other stimulants including caffeine and multiple drug use and the use of other psychoactive substances in the total population and in the 20–29 age group, and to alcohol, cannabinoids, and other stimulants including caffeine in the 30–39 age group. Compared to the pre-pandemic period, the COVID-19 pandemic was associated with a significantly decreased risk of opioid disorders in the total population and in the 30–39 age group, and with alcohol in the 20–29 and 50–59 age groups. Diagnoses related to alcohol accounted for the highest proportion in each age group in both males and females during both the pre-pandemic and pandemic periods.

In addition, specific trends in only males indicated that the pandemic was associated with a significantly increased risk of multiple drug use and the use of other psychoactive substances in the ≤19 age group; with volatile solvents in the 30–39 age group; with other stimulants including caffeine and multiple drug use and the use of other psychoactive substances in the 40–49 age group; and with cannabinoids and multiple drug use and the use of other psychoactive substances in the 50–59 and ≥60 age groups. Among males, the pandemic was associated with significantly decreased risks of disorders related to cannabinoids in the total group of males and the 20–29 age group, opioids in the 40–49 age group, and alcohol in the ≥60 age group. The trends in females were that the pandemic was associated with a significantly increased risk of an opioid-related disorder in the 50–59 age group and a significantly decreased risk of a cannabinoid-related disorder in the ≤19 age group.

Thus, for younger people in Kazakhstan, the COVID-19 pandemic was accompanied by an increased risk of diagnoses of disorders associated with other stimulants including caffeine and multiple drug use and the use of other psychoactive substances. For the 30–39 age group, the pandemic was associated with a lower risk of opioid-related disorder among both males and females. In addition, the risk of alcohol-related mental or behavioral disorders was increased at the 30-year-old level and decreased at the 20- and 50-year-old levels. During the pandemic, disorders associated with multiple drug use and the use of other psychoactive substances were increased in almost all of the age groups among males, and cannabinoid-related disorders were increased among males aged ≥50 years. Our results demonstrate that alcohol use was the most likely to be associated with mental and behavioral disorders due to psychoactive substance use in each age group in both males and females.

It has been proposed that the increased risks for multiple drug use and the use of other psychoactive substances during the COVID-19 years were based on changes in individuals’ lifestyles due to staying at home/restrictions on going outside, loneliness, and stress from economic hardships that affected respondents’ mental state, leading them to use multiple drugs and seek pleasure from psychoactive substances [41,42]. The respondents in those studies also indicated they also spent more time online during the pandemic, which may have contributed to an increased ability to obtain drugs online. As other factors, the impact of the COVID-19 on supply chains and markets during the pandemic has been suggested to affect the availability of certain substances for substance users [43,44], and a clear effect of socioeconomic status on COVID-19 mortality was observed in a 25- to 64-year-old population [45]. In addition, cultural factors that vary in some countries may also have an impact.

The reason(s) for the increased risk of the use of other stimulants including caffeine were presumably the same as or similar to those mentioned above for multiple drug use and the use of other psychoactive substances. Sedatives, hypnotics, anxiolytics, and caffeine are included in this category, and these are most likely to be prescribed by a medical facility or obtained online. The continual overuse of these drugs increases the likelihood of requiring hospitalization and involves a non-zero chance of death [46]. The intake of caffeine from coffee and energy drinks during and after the COVID-19 lockdowns was reported to be decreased compared to the intake prior to COVID-19 [47]. That said, many people felt the need to increase their consumption of coffee, a stimulant beverage, after the start of the COVID-19 lockdowns [48]

Opioids are generally used for medical purposes [49], as their primary purpose is to relieve pain. A review conducted in the U.S. during COVID-19 included the mortality rate due to opioid-related overdoses [50].

A detailed analysis of alcohol overdoses in the U.S., India, Canada, and Iran during the COVID-19 pandemic revealed that most of the overdoses were among males and that the causes among all age groups were related to socioeconomic changes, interruptions in mental health services, and physical isolation [51]. Among adult females in the U.S., the COVID-19 pandemic was significantly associated with increased alcohol use [52].

A study of cannabinoids indicated that cannabis was the most frequently detected drug in both hashish and marijuana seizures [53]. The mental and physical effects of cannabis use disorder may become evident, and acute intoxication and chronic symptoms may develop [52,54,55].

Discrepant findings regarding which sex was more likely to abuse or depend on substance use during COVID-19 have been obtained [51,56,57,58]. Our present analyses revealed that, in Kazakhstan, the effects of mental and behavioral disorders due to psychoactive substance use during COVID-19 occurred in both males and females, and these effects tended to be more evident in males than in females. The most important aspect of our findings is that, as a form of primary prevention, people need to properly understand the risks of mental and behavioral disorders due to psychoactive substance use, and people need to be apprised of the risks of using illicit substances that have recently come to the fore. Secondary and tertiary prevention is also important; continuous support from psychiatric facilities is needed, and further support for forms of healthcare other than drug therapy is necessary. In Kazakhstan, public education concerning the prevention of substance use continues, but its content is poor, and new public health content during the pandemic was lacking. Recommendations for preventive measures, treatment strategies, and community-based interventions are essential for mitigating the impact of substance use disorders on public health. These considerations should also be considered in various areas, including public health [59,60,61]. Personnel in medicine and nursing, the government, private organizations, and the public need to work together to respond to substance abuse over the long term.

There are study limitations to consider. We focused mainly on the ICD disorders F10 to F19 (excluding F17) in Kazakhstan, and details for each code were not available. Our exclusion of data concerning disorders related to tobacco use (due to the lack of information prior to a specific timepoint) may have introduced bias, as tobacco use is a significant factor influencing substance use patterns and could potentially impact the study outcomes. Another limitation is the unavailability of data regarding important confounding factors such as socioeconomic status, access to healthcare, and other contextual variables that may affect mental and behavioral health outcomes. These confounding factors could potentially influence the observed associations between the pandemic and mental and behavioral disorders due to substance use. As a result, our findings should be interpreted with caution. Future studies should integrate these variables to obtain a more nuanced understanding of the impact of the COVID-19 pandemic.

However, the contents of this study regarding mental and behavioral disorders due to psychoactive substance use and the pandemic in Kazakhstan are new and valuable. The impacts of the pandemic have varied among countries, and not all of the related issues are uniform. However, it is very important that each country conducts thorough reviews based on their respective data.

## 5. Conclusions

The results of our retrospective analyses demonstrated that the COVID-19 pandemic had significant effects on individuals in Kazakhstan with mental and behavioral disorders due to psychoactive substance use, with differences according to sex and age. The causes and consequences of these effects require further investigation based on individual countries, not only in Kazakhstan but also in many middle- and low-income countries, in order to develop preventive interventions for persons with mental and behavioral disorders during further global crises like the coronavirus pandemic. Joint global and international research on this issue is also merited.

## Figures and Tables

**Table 1:** Comparison of mental and behavioral disorders due to substance use in Kazakhstan during the pre-pandemic and COVID-19 pandemic periods in each age group of the overall group.

Mental and Behavioral Disorders Due to Psychoactive Substance Use (ICD-10 Code)	2018–2019 (Pre-Pandemic), n	2020–2021 (Pandemic), n	OR (95%CI)
**Age ≤ 19 years:**			
Alcohol (F10)	889	592	0.936 (0.795–1.102)
Opioids (F11)	45	41	1.346 (0.871–2.074)
Cannabinoids	290	162	0.781 (0.632–0.965) *
Sedatives and hypnotics (F13)	0	2	(–)
Cocaine (F14)	0	0	(–)
Other stimulants including caffeine (F15)	19	27	2.101 (1.165–3.867) *
Hallucinogens (F16)	6	1	0.271 (0.010–1.649)
Volatile solvents (F18)	159	115	1.065 (0.824–1.373)
Multiple drug use and the use of other psychoactive substances (F19)	64	67	1.568 (1.101–2.234) *
Total	1472	1007	
**Age 20–29 years:**			
Alcohol (F10)	11,872	8343	0.925 (0.886–0.965) **
Opioids (F11)	1041	806	1.070 (0.973–1.176)
Cannabinoids (F12)	5617	3848	0.923 (0.880–0.968) **
Sedatives and hypnotics (F13)	4	5	1.708 (0.436–7.206)
Cocaine (F14)	0	0	(–)
Other stimulants including caffeine (F15)	242	401	2.317 (1.974–2.725) †
Hallucinogens (F16)	0	1	(–)
Volatile solvents (F18)	175	152	1.198 (0.962–1.490)
Multiple drug use and the use of other psychoactive substances (F19)	1163	1051	1.263 (1.159–1.377) †
Total	20,114	14,607	
**Age 30–39 years:**			
Alcohol (F10)	50,186	43,302	1.056 (1.029–1.084) †
Opioids (F11)	7251	4566	0.717 (0.690–0.745) †
Cannabinoids (F12)	5544	5321	1.141 (1.097–1.186) †
Sedatives and hypnotics (F13)	12	4	0.402 (0.109–1.170)
Cocaine (F14)	0	0	(–)
Other stimulants including caffeine (F15)	173	456	3.111 (2.616–3.717) †
Hallucinogens (F16)	2	2	1.175 (0.1221–1.283)
Volatile solvents (F18)	82	97	1.390 (1.036–1.869) *
Multiple drug use and the use of other psychoactive substances (F19)	3755	3291	1.031 (0.983–1.082)
Total	67,005	57,039	
**Age 40–49 years:**			
Alcohol (F10)	64,005	60,369	1.005 (0.980–1.032)
Opioids (F11)	8100	7221	0.940 (0.909–0.972) **
Cannabinoids (F12)	2796	2637	1.001 (0.948–1.057)
Sedatives and hypnotics (F13)	17	11	0.690 (0.311–1.466)
Cocaine (F14)	0	0	(–)
Other stimulants including caffeine (F15)	36	90	2.648 (1.814–3.948) †
Hallucinogens (F16)	0	0	(–)
Volatile solvents (F18)	11	13	1.251 (0.555–2.876)
Multiple drug use and the use of other psychoactive substances (F19)	3418	3517	1.097 (1.045–1.151) **
Total	78,383	73,858	
**Age 50–59 years:**			
Alcohol (F10)	48,904	45,660	0.894 (0.855–0.935) †
Opioids (F11)	2032	2057	1.078 (1.012–1.147) *
Cannabinoids (F12)	1022	1101	1.147 (1.052–1.250) **
Sedatives and hypnotics (F13)	8	4	0.541 (0.139–1.755)
Cocaine (F14)	0	0	(–)
Other stimulants including caffeine (F15)	2	3	1.555 (0.237–13.391)
Hallucinogens (F16)	0	0	(–)
Volatile solvents (F18)	1	1	1.061 (0.0272–41.398)
Multiple drug use and the use of other psychoactive substances (F19)	969	1047	1.150 (1.053–1.256) **
Total	52,938	49,873	
**Age ≥60 years:**			
Alcohol (F10)	26,115	28,857	0.860 (0.778–0.951) **
Opioids (F11)	327	365	1.006 (0.866–1.169)
Cannabinoids (F12)	204	288	1.275 (1.065–1.528) **
Sedatives and hypnotics (F13)	12	9	0.679 (0.274–1.620)
Cocaine (F14)	0	0	(–)
Other stimulants including caffeine (F15)	0	1	(–)
Hallucinogens (F16)	0	0	(–)
Volatile solvents (F18)	0	0	(–)
Multiple drug use and the use of other psychoactive substances (F19)	152	230	1.366 (1.113–1.681) **
Total	26,810	29,750	
**All ages:**			
Alcohol (F10)	201,971	186,137	1.059 (1.043–1.075) †
Opioids (F11)	187,96	15,041	0.868 (0.849–0.888) †
Cannabinoids (F12)	15,473	13,319	0.940 (0.918–0.963) †
Sedatives and hypnotics (F13)	53	36	0.746 (0.484–1.135)
Cocaine (F14)	0	0	(–)
Other stimulants including caffeine (F15)	472	977	2.274 (2.038–2.540) †
Hallucinogens (F16)	8	4	0.559 (0.144–1.812)
Volatile solvents (F18)	428	378	0.968 (0.843–1.112)
Multiple drug use and the use of other psychoactive substances (F19)	9521	9193	1.061 (1.030–1.092) †
Total	246,722	225,085	

The numbers of new diagnoses during each period are presented. Odds ratios (OR) were calculated using the number of new diagnoses in the pandemic period compared to the pre-pandemic period. OR > 1 indicates an increase in the likelihood of new diagnoses in the pandemic period, and OR < 1 indicates a decrease. The 95% confidence intervals (CIs) indicate the precision of the OR values. * *p* < 0.05, ** *p* < 0.01, † *p* < 0.0001.

**Table 2:** Comparison of mental and behavioral disorders due to substance use in Kazakhstan during the pre-pandemic and COVID-19 pandemic periods in each age group of males.

Mental and Behavioral Disorders Due to Psychoactive Substance Use (ICD-10 Code)	2018–2019 (Pre-Pandemic), n	2020–2021 (Pandemic), n	OR (95%CI)
**Age ≤** **19 years:**			
Alcohol (F10)	645	401	0.900 (0.748–1.084)
Opioids (F11)	34	28	1.275 (0.760–1.094)
Cannabinoids (F12)	264	155	0.875 (0.698–1.094)
Sedatives and hypnotics (F13)	0	1	(–)
Cocaine (F14)	0	0	(–)
Other stimulants including caffeine (F15)	12	15	1.930 (0.894–4.263)
Hallucinogens (F16)	6	1	0.284 (0.011–1.730)
Volatile solvents (F18)	142	99	1.081 (0.819–1.421)
Multiple drug use and use of other psychoactive substances (F19)	46	49	1.678 (1.108–2.545) *
Total	1149	749	
**Age 20–29 years:**			
Alcohol (F10)	10,123	7091	0.948 (0.906–0.992) *
Opioids (F11)	873	659	1.055 (0.951–1.171)
Cannabinoids (F12)	5526	3761	0.928 (0.883–0.975) **
Sedatives and hypnotics (F13)	3	5	2.278 (0.537–11.815)
Cocaine (F14)	0	0	(–)
Other stimulants including caffeine (F15)	181	284	2.214 (1.837–2.675) †
Hallucinogens (F16)	0	1	(–)
Volatile solvents (F18)	161	140	1.215 (0.967–1.525)
Multiple drug use and use of other psychoactive substances (F19)	1054	912	1.222 (1.115–1.340) †
Total	17,921	12,853	
**Age 30–39 years:**			
Alcohol (F10)	41,951	36,284	1.052 (1.023–1.081) **
Opioids (F11)	6316	3975	0.714 (0.685–0.745) †
Cannabinoids (F12)	5474	5239	1.136 (1.091–1.182) †
Sedatives and hypnotics (F13)	6	3	0.601 (0.120–2.353)
Cocaine (F14)	0	0	(–)
Other stimulants including caffeine (F15)	139	384	3.252 (2.685–3.963) †
Hallucinogens (F16)	2	2	1.172 (0.122–11.255)
Volatile solvents (F18)	74	89	1.409 (1.036–1.924) *
Multiple drug use and use of other psychoactive substances (F19)	3477	3045	1.028 (0.977–1.081)
Total	57,439	49,021	
**Age 40–49 years:**			
Alcohol (F10)	52,934	49,966	1.010 (0.982–1.038)
Opioids (F11)	7213	6395	0.934 (0.902–0.968) **
Cannabinoids (F12)	2767	2601	0.998 (0.945–1.054)
Sedatives and hypnotics (F13)	10	3	0.330 (0.071–1.098)
Cocaine (F14)	0	0	(–)
Other stimulants including caffeine (F15)	31	78	2.664 (1.775–4.102) †
Hallucinogens (F16)	0	0	(–)
Volatile solvents (F18)	9	11	1.293 (0.529–3.247)
Multiple drug use and use of other psychoactive substances (F19)	3158	3241	1.094 (1.041–1.151) **
Total	66,122	62,295	
**Age 50–59 years:**			
Alcohol (F10)	40,630	37,915	0.901 (0.860 0.945) †
Opioids (F11)	1823	1806	1.054 (0.986–1.127)
Cannabinoids (F12)	1010	1087	1.147 (1.051–1.250) **
Sedatives and hypnotics (F13)	0	2	(–)
Cocaine (F14)	0	0	(–)
Other stimulants including caffeine (F15)	2	3	1.556 (0.237–13.395)
Hallucinogens (F16)	0	0	(–)
Volatile solvents (F18)	1	1	1.062 (0.027–41.411)
Multiple drug use and use of other psychoactive substances (F19)	913	982	1.145 (1.046–1.255) **
Total	44,379	41,796	
**Age ≥60 years:**			
Alcohol (F10)	21,434	23,665	0.857 (0.771–0.953) **
Opioids (F11)	287	315	0.989 (0.842–1.162)
Cannabinoids (F12)	200	284	1.283 (1.070–1.540) **
Sedatives and hypnotics (F13)	1	4	3.270 (0.453–89.245)
Cocaine (F14)	0	0	(–)
Other stimulants including caffeine (F15)	0	1	(–)
Hallucinogens (F16)	0	0	(–)
Volatile solvents (F18)	0	0	(–)
Multiple drug use and use of other psychoactive substances (F19)	134	197	1.327 (1.066–1.658) *
Total	22,056	24,466	
**All ages:**			
Alcohol (F10)	167,717	154,494	1.064 (1.047–1.081) †
Opioids (F11)	16,546	13,167	0.865 (0.845–0.886) †
Cannabinoids (F12)	15,241	13,090	0.939 (0.917–0.962) †
Sedatives and hypnotics (F13)	20	18	0.990 (0.517–1.882)
Cocaine (F14)	0	0	(–)
Other stimulants including caffeine (F15)	365	764	2.304 (2.036–2.613) †
Hallucinogens (F16)	8	4	0.560 (0.144–1.817)
Volatile solvents (F18)	387	340	0.965 (0.834–1.117)
Multiple drug use and use of other psychoactive substances (F19)	8782	8416	1.055 (1.024–1.088) **
Total	209,066	190,293	

The numbers of new diagnoses during each period are presented. Odds ratios (OR) were calculated using the number of new diagnoses in the pandemic period compared to the pre-pandemic period. OR > 1 indicates an increase in the likelihood of new diagnoses in the pandemic period, and OR < 1 indicates a decrease. The 95% confidence intervals (CIs) indicate the precision of the OR values. * *p* < 0.05, ** *p* < 0.01, † *p* < 0.0001.

**Table 3:** Comparison of mental and behavioral disorders due to substance use in Kazakhstan during the pre-pandemic and COVID-19 pandemic periods in each age group of females.

Mental and Behavioral Disorders Due to Psychoactive Substance Use (ICD-10 Code)	2018–2019 (Pre-Pandemic), n	2020–2021 (Pandemic), n	OR (95%CI)
**Age ≤** **19 years:**			
Alcohol (F10)	244	191	0.923 (0.633–1.348)
Opioids (F11)	11	13	1.501 (0.654–3.505)
Cannabinoids (F12)	26	7	0.324 (0.127–0.725) **
Sedatives and hypnotics (F13)	0	1	(–)
Cocaine (F14)	0	0	(–)
Other stimulants including caffeine (F15)	7	12	2.179 (0.854–6.025)
Hallucinogens (F16)	0	0	(–)
Volatile solvents (F18)	17	16	1.190 (0.581–2.427)
Multiple drug use and the use of other psychoactive substances (F19)	18	18	1.270 (0.640–2.520)
Total	323	258	
**Age 20–29 years:**			
Alcohol (F10)	1749	1252	0.633 (0.547–0.733) †
Opioids (F11)	168	147	1.103 (0.875–1.389)
Cannabinoids (F12)	91	87	1.206 (0.891–1.630)
Sedatives and hypnotics (F13)	1	0	(–)
Cocaine (F14)	0	0	(–)
Other stimulants including caffeine (F15)	61	117	2.494 (1.825–3.442) †
Hallucinogens (F16)	0	0	(–)
Volatile solvents (F18)	14	12	1.074 (0.483–2.350)
Multiple drug use and the use of other psychoactive substances (F19)	109	139	1.645 (1.270–2.135) **
Total	2193	1754	
**Age 30–39 years:**			
Alcohol (F10)	8235	7018	1.134 (1.039–1.239) **
Opioids (F11)	935	591	0.735 (0.660–0.818) †
Cannabinoids (F12)	70	82	1.401 (1.017–1.935) *
Sedatives and hypnotics (F13)	6	1	0.222 (0.009–1.346)
Cocaine (F14)	0	0	(–)
Other stimulants including caffeine (F15)	34	72	2.534 (1.696–3.862) †
Hallucinogens (F16)	0	0	(–)
Volatile solvents (F18)	8	8	1.193 (0.432–3.297)
Multiple drug use and the use of other psychoactive substances (F19)	278	246	1.058 (0.888–1.259)
Total	9566	8018	
**Age 40–49 years:**			
Alcohol (F10)	11,071	10,403	0.964 (0.885–1.050)
Opioids (F11)	887	826	0.986 (0.894–1.088)
Cannabinoids (F12)	29	36	1.316 (0.806–2.166)
Sedatives and hypnotics (F13)	7	8	1.209 (0.426–3.512)
Cocaine (F14)	0	0	(–)
Other stimulants including caffeine (F15)	5	12	2.500 (0.915–8.038)
Hallucinogens (F16)	0	0	(–)
Volatile solvents (F18)	2	2	1.060 (0.110–10.186)
Multiple drug use and the use of other psychoactive substances (F19)	260	276	1.129 (0.951–1.340)
Total	12,261	11,563	
**Age 50–59 years:**			
Alcohol (F10)	8274	7745	0.804 (0.684–0.944) **
Opioids (F11)	209	251	1.281 (1.064–1.544) **
Cannabinoids (F12)	12	14	1.234 (0.565–2.738)
Sedatives and hypnotics (F13)	8	2	0.280 (0.038–1.145)
Cocaine (F14)	0	0	(–)
Other stimulants including caffeine (F15)	0	0	(–)
Hallucinogens (F16)	0	0	(–)
Volatile solvents (F18)	0	0	(–)
Multiple drug use and the use of other psychoactive substances (F19)	56	65	1.231 (0.860–1.768)
Total	8559	8077	
**Age ≥60 years:**			
Alcohol (F10)	4681	5192	0.881 (0.644–1.199)
Opioids (F11)	40	50	1.125 (0.741–1.718)
Cannabinoids (F12)	4	4	0.900 (0.203–3.990)
Sedatives and hypnotics (F13)	11	5	0.415 (0.128–1.159)
Cocaine (F14)	0	0	(–)
Other stimulants including caffeine (F15)	0	0	(–)
Hallucinogens (F16)	0	0	(–)
Volatile solvents (F18)	0	0	(–)
Multiple drug use and the use of other psychoactive substances (F19)	18	33	1.647 (0.935–2.998)
Total	4754	5284	
**All ages:**			
Alcohol (F10)	34,254	31,643	0.998 (0.949–1.050)
Opioids (F11)	2250	1874	0.896 (0.841–0.954) **
Cannabinoids (F12)	232	229	1.069 (0.890–1.284)
Sedatives and hypnotics (F13)	33	18	0.593 (0.326–1.043)
Cocaine (F14)	0	0	(–)
Other stimulants including caffeine (F15)	107	213	2.160 (1.716–2.735) †
Hallucinogens (F16)	0	0	(–)
Volatile solvents (F18)	41	38	1.003 (0.642–1.564)
Multiple drug use and the use of other psychoactive substances (F19)	739	777	1.141 (1.031–1.263) *
Total	37,656	34,792	

The numbers of new diagnoses during each period are presented. Odds ratios (OR) were calculated using the number of new diagnoses in the pandemic period compared to the pre-pandemic period. OR > 1 indicates an increase in the likelihood of new diagnoses in the pandemic period, and OR < 1 indicates a decrease. The 95% confidence intervals (CIs) indicate the precision of the OR values. * *p* < 0.05, ** *p* < 0.01, † *p* < 0.0001.

## Data Availability

Data are contained within the article.
